# Photon-counting CT-angiography in comparison to digital subtraction angiography and MR-angiography for assessing intra-aneurysmal flow disrupting devices

**DOI:** 10.1007/s13760-026-03112-x

**Published:** 2026-06-30

**Authors:** Frederic De Beukelaer, Laura L. Wuyts, Martin Wiesmann, Hani Ridwan, Steven Van Hedent, Charlotte S. Weyland

**Affiliations:** 1 Department of Neuroradiology, University Hospital RWTH Aachen, Pauwelsstraße 30, 52074 Aachen, Germany; 2 Department of Radiology, AZ Sint-Lucas, Gent, Belgium

**Keywords:** Cerebral aneurysm, CT-Angiography, Intraaneurysmal flow disrupting device, Digital subtraction angiography, Photon-counting CT

## Abstract

**Purpose:**

This study aimed to evaluate the potential of Photon-Counting Detector CT Angiography (PCD-CTA) for the post-interventional assessment of intraaneurysmal flow disrupting devices (IAFD).

**Methods:**

This retrospective analysis evaluates consecutive patients with intracranial aneurysms treated with IAFD between April 2023 and April 2025, who underwent PCD-CTA, MR angiography and DSA as part of their clinical diagnostic work-up routine. Polyenergetic images, iodine and virtual monoenergetic imaging reconstructions with different keV levels (40 and 80) and with different Head vessel kernels (Hv56 and Hv72) were acquired with and without iterative metal artifact reduction. Three independent readers assessed image quality using a 5-point Likert scale and region of interest analysis. The different kernels, keV and the optimized spectral reconstructions were compared in descriptive analysis.

**Results:**

A total of 10 patients (mean age 60.41 ± 11.43 (43–74) years; 6 women) with intracranial aneurysms treated with either Contour device (*n* = 6) or WEB device (*n* = 4) were included. Reconstructions using Hv56 and a 40 keV yielded increased signal and contrast to noise ratios and were preferred for visualizing the parent vessel lumen compared to Hv72 and higher keV levels (*p* < 0.001). Assessing the different spectral reconstructions virtual monoenergetic reconstructions proved to be best to evaluate the parent vessel lumen (*p* = 0.040).

**Conclusion:**

Spectral reconstructions of PCD-CTA with a smoother reconstruction kernel and a low keV level seem to be beneficial to achieve optimal image quality for the diagnostic evaluation of patients with intracranial IAFD.

**Supplementary Information:**

The online version contains supplementary material available at https://doi.org/10.1007/s13760-026-03112-x.

## Introduction

In clinical practice, imaging protocols to assess occlusion rate, aneurysm occlusion, and parent vessel pathologies following intra-aneurysmal flow disrupting devices (IAFD) placement vary among centers [1].

Based on current published literature Magnetic Resonance Angiography (MRA) seems to be a common non-invasive follow-up imaging modality for WEB devices, while its value is more limited for evaluation of Contour devices [2–4].

While a single study supported Energy-integrating CT Angiography (EID CTA) for follow-up of WEB-Devices, this modality is susceptible to beam-hardening artifacts [4, 5].

However in-vitro evaluations of IAFD using spectral CT showed promising results for improving diagnostic CT performance [4].

Photon-counting-Detector CT-Angiography (PCD-CTA) provides high spatial resolution and spectral reconstructions with promising results in evaluating intracranial vessels after implantation of neurovascular devices [6, 7].

We aimed to assess the imaging post-processing possibilities of PCD-CTA to find the best reconstruction kernels and keV-levels for the evaluation of IAFD in intracranial aneurysms. Further we investigated the diagnostic accuracy of PCD-CTA compared to DSA and MRA.

## Materials and methods

This study is a single-center, retrospective analysis of consecutive patients treated for intracranial aneurysms using IAFD between April 2023 and November 2024.

The study was approved by the Medical Faculty at University Hospital RWTH Aachen, Aachen, Germany, under the registration number EK 24-270. Patient consent was waived due to the retrospective nature of the study design. The study is reported in adherence to the STROBE criteria [8].

All patients were scanned with a dual-source photon-counting CT scanner (NAEOTOM Alpha, Siemens Healthineers, Erlangen, Germany, software version syngo CT VB10) operated in ultra-high-resolution mode. The imaging protocol consisted of contrast-enhanced PCD-CTA, centered on the implanted device, and triggered by bolus tracking.

**Fig. 1:**
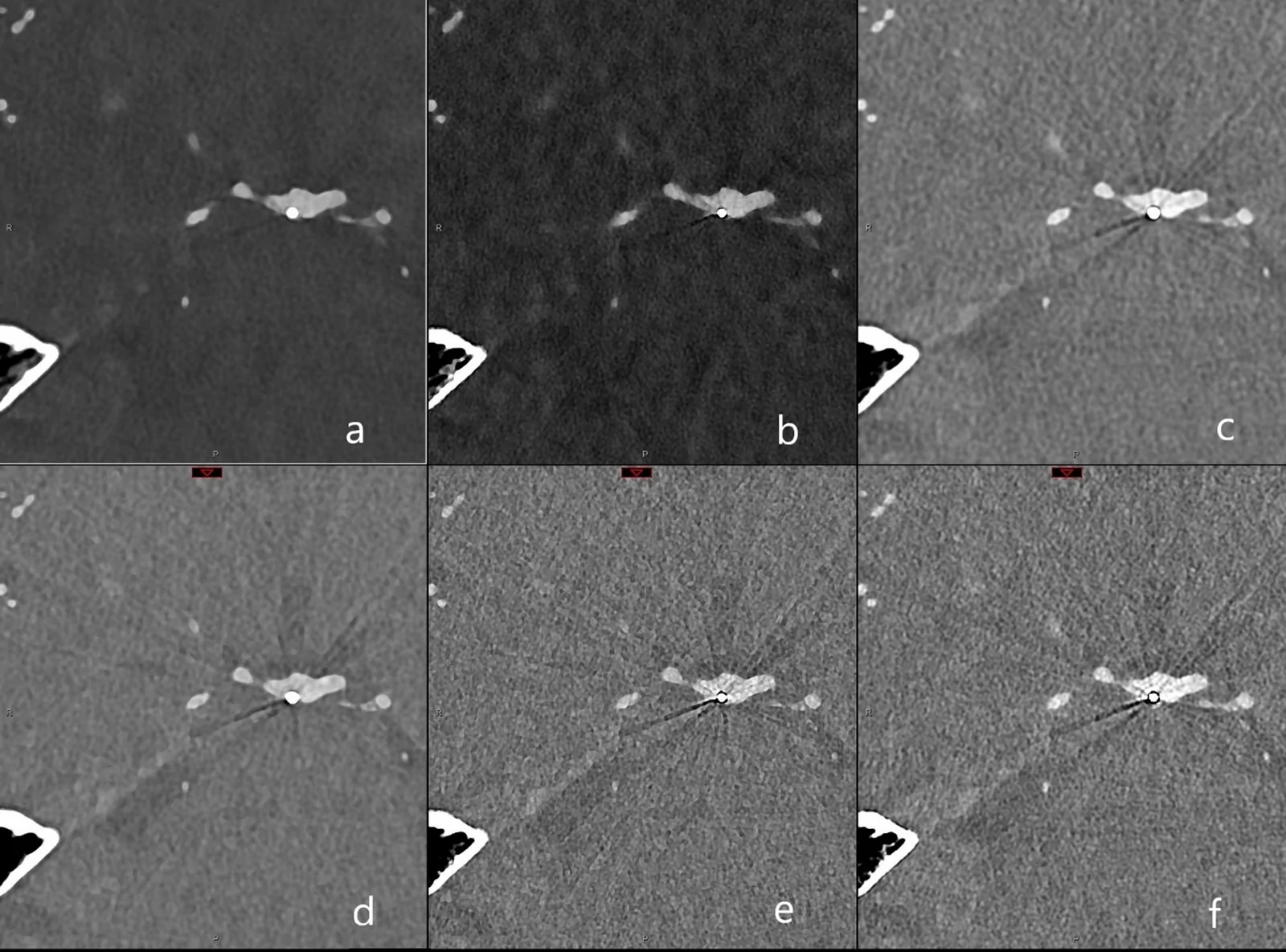
Illustration of reconstruction protocol for Polyenergetic (PE) and virtual mono energetic imaging reconstructions (VMI) with two different kernels. In the first row VMI and in the second row PE are listed and in the three columns. In detail: **a** = Hv 56, 40 keV, VMI; **b** = Hv 56, 80 keV VMI; **c** = Hv 72, 40 keV, VMI; **d** = Hv56, PE; **e** = Hv 72, PE; **f** = Hv72 80 keV, VMI. Hv: Head vascular kernel

In all patients polyenergetic (PE) and iodine reconstructions (IOD) were performed with two different head vessel (Hv) kernels (Hv56 and Hv72), virtual monoenergetic imaging (VMI) reconstructions were performed using Hv56 and 72 with two different kiloelectron Volt (keV) levels (40 and 80 keV) as visualized in Fig. 1.

Detailed information on the PCD-CT image acquisition and post-processing, including spectral reconstruction specifics, can be found in the supplementary data.

### Subjective image quality assessment

Three radiologists with 10 years (FDB), 11 years (LW) and 12 years (SVH) of experience independently evaluated all images using the syngo.via workstation (version VB10A, Siemens Healthineers) while remaining blinded to clinical data, as well as DSA and MRI findings. Image quality was rated on a 5-point Likert scale (1 = “non-diagnostic,” 5 = “excellent”) based on lumen visibility of the parent vessel, as well as regions with maximum artifacts.

### Objective image quality assessment

Three radiologists independently placed four regions of interest (ROI) of identical diameter in each reconstruction: one in the parent vessel within 1 cm proximal to the IAFD, one in the vessel adjacent to the IAFD, one in the air above the calvarium, and one in the superficial temporal muscle. The mean Hounsfield units (HU) were recorded from these ROIs.

SNR (signal-to-noise ratio) and CNR (contrast-to-noise ratio) were calculated as follows:

SNR = signal_artery_/standard deviation_artery_.

CNR = (signal_artery_ – signal_muscle_)/standard deviation_air_.

### Assessment of aneurysm occlusion using PCD-CTA, MRA and DSA

Two board certified interventional neuroradiologists (CW and FDB) independently assessed aneurysm occlusion and the parent vessel in PCD-CTA, MRA and DSA at three different time points separated by a week.

Segments scored as non-diagnostic due to impaired image quality on all reconstructed CT and MR images were rated as potential sites of residual aneurysms.

### DSA as the reference standard measurement

DSA was performed using standard techniques by board-certified interventional neuroradiologists. At least two projections were obtained, followed by an independent review by two readers blinded to clinical data, especially the DSA, CTA and MRA reports.

### Radiation dose assessment

Radiation dose parameters, including dose length product (DLP) and dose area product (DAP) were extracted from the CT and DSA reports.

### Statistical analysis

IBM SPSS Statistics software (version 28.0) was used for statistical analysis.

Normality of distributions for descriptive analysis was assessed using the Shapiro-Wilk test. Quantitative variables were expressed as mean ± standard deviation (SD) or median and interquartile ranges (IQR). Likert scores were pooled across readers.

Friedman’s test was used to compare different kernels for each reconstruction type (IOD, PE, VMI), with pairwise comparisons. In case of the VMI, the two keV levels were compared for each kernel before using the test. Friedman’s test was used to compare the Likert values of the different optimized spectral reconstructions (IOD, PE, VMI).

Interobserver agreement was expressed in Cohen’s kappa (κ) value and interpreted as follows: ≤ 0.20 as none, 0.21–0.40 as fair, 0.41–0.60 as moderate, 0.61–0.80 as substantial, and ≥ 0.81 as almost perfect agreement [9]. All p-values were corrected for multiple comparisons using the Bonferroni correction. A 95% confidence interval range (CI) was calculated for diagnostic accuracy tests, and a two-tailed p-value of < 0.05 was considered statistically significant.

Diagnostic performance of PCD-CTA compared with the reference standard of DSA was evaluated using the pooled readers’ assessment of the two vessel sites in 10 patients. Receiver operating characteristic (ROC) curve analysis was performed to calculate the area under the curve (AUC). Non-parametric distribution assumptions were made for standard error approximation. If the parent vessel could not be adequately assessed, a residual aneurysm was assumed.

Sensitivity, specificity, positive predictive value (PPV), and negative predictive value (NPV) were calculated for detecting an aneurysm remnant, classified following the criteria of the Raymond-Roy Occlusion Classification [10].

## Results

Patient characteristics are detailed in Table 1.

**Table 1:** Patient characteristics

Age (years)*	60.41 ± 11.43
Sex (female/male)	7/3
IAFD (WEB/Contour)	4/6
*Aneurysm Locations*	
Internal cerebral artery	4 (40.0)
Posterior communicating artery	1 (10.0)
Anterior communicating artery	1 (10.0)
Basilar artery	3 (30.0)
Medial cerebral artery	1 (10.0)
Aneurysm size (mm)*	7.21 ± 1.92
DLP mGycm*	147.31 ± 42.42
DAP µGycm^2^*	1334.63 ± 526.71

Except where indicated, data are numbers of participants, with percentages in parentheses

DAP: Dose area product, DLP: Dose length product, IAFD: Intraaneurysmal flow disrupting device, mGycm: Milligray centimeter, mGycm^2^: Milligray square centimeter, mm: millimeter

*Data are means ± standard deviations

A total of 10 patients (mean age 60.4 ± 11.4 (43–74) years; 7 women) with intracranial aneurysms treated with either Contour (6) or WEB devices (4) were included.

### Objective evaluation of image quality

Of the 10 patients, 20 vessel segments were evaluated, in three reconstructions (IOD, PE, VMI).

#### Virtual monoenergetic imaging reconstructions (VMI)

VMI reconstructions showed a decrease in SNR and CNR across different kernel and keV levels.

**Fig. 2:**
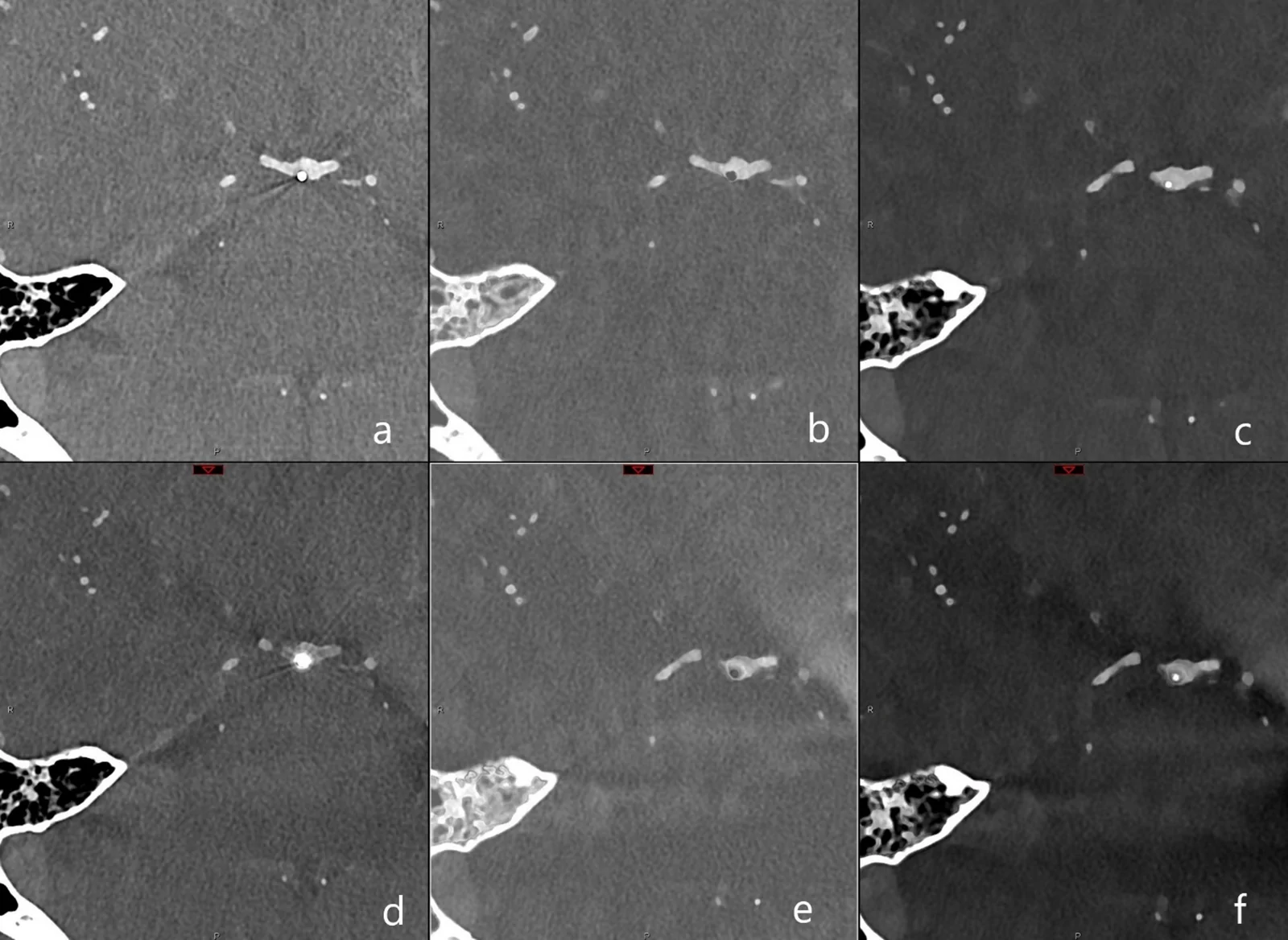
Illustration of reconstruction protocol for Polyenergetic (PE), iodine (IOD) and virtual mono energetic imaging (VMI) reconstructions with and without iMAR. In the first row PE, IOD and VMI without iMAR and in the second row with iMAR are listed and in the three columns. In detail: **a** = Hv 56, PE; **b** = Hv 56, IOD; **c** = Hv 56 at 40 keV VMI; **d** = Hv56, PE with iMAR; **e** = Hv 56 IOD with iMAR, IOD; **f** = Hv56 VMI with iMAR

1. In VMI, the lowest keV-level (40 keV) achieved the highest SNRs and CNRs, significantly outperforming the higher keV level (80 keV). For example, SNR for Hv56 at 40 keV was higher than at 80 keV in the parent vessel (mean ± standard deviation (SD): 31.7 vs. 16.0 ± 14.3, *p* = 0.010), as well as in the vessel at the IAFD site (median (IQR): 13.6 (15.2) vs. 6.8 (6.1), *p* < 0.001).
2. Smoother kernels (Hv56) resulted in higher SNRs and CNRs compared to harder kernels (Hv72) at 40 keV. As an example, SNR values for Hv56 and Hv72 kernels for 40 keV were in the parent vessel (31.6 ± 10.6 vs. 17.6 ± 7.1 (mean ± SD), *p* < 0.001), in the vessel at the IAFD site (11.6 ± 10.9 vs. 8.9 ± 7.1 (mean ± SD), *p* = 0.030).
3. Reconstructions with iMAR did not improve SNR and CNR in the parent vessel as visualized in Fig. 2. As an example, SNR values for Hv56 and Hv56 with iMAR for 40 keV were in the intracranial parent vessel (31.7 ± 10.6 (mean ± SD) vs. 25.0 ± 15.4 (mean ± SD), *p* = 0.001).

However, SNR in the vessel at the IAFD site differed in favor of reconstructions without iMAR (11.6 ± 10.9 (mean ± SD) vs. 7.6 (20.6) (median (IQR), *p* = 0.001).

#### Iodine and polyenergetic reconstructions

For iodine and polyenergetic reconstructions, smoother kernels consistently provided the highest SNRs and CNRs in the parent vessel and in the vessel at the IAFD site. As an example, SNR values for Hv56 and Hv72 kernels were in the intracranial parent vessel (14.6 ± 2.8 (mean ± SD) vs. 8.5 ± 2.7 (mean ± SD), *p* < 0.001), in the vessel at the IAFD site (7.8 (8.9) (median (IQR)) vs. 4.8 (7.7) (median (IQR)): *p* < 0.001).

#### Comparison of Likert scales between the two different IAFD

The Likert scale distribution for most reconstructions was similar between the two IAFDs. Although individual reconstructions received better ratings in one group, e.g. iodine reconstruction with Hv72 was rated better in patients with Contour device (3 (2) (median (IQR))) than in patients with WEB-device (2 (2), *p* = 0.025), VMI reconstruction with Hv56 and 40 keV received the best ratings in both subgroups (Contour group: 4 (2) (median (IQR) and WEB-device group: 4 (3)).

### Objective evaluation of image quality

For VMI, images reconstructed at 40 keV achieved the highest score (*p* < 0.001). For the parent vessel images reconstructed with Hv56, Hv72 at 40 keV with and without iMAR achieved comparable results (*p* = 0.120).

At the IAFD site, polyenergetic and iodine reconstructions with iMAR (Hv56) were unexpectedly rated significantly lower than those without iMAR. For example, iodine re-constructions with Hv56 and iMAR received a median score of 2, while Hv56 without iMAR had a median score of 4 (*p* = 0.001).

The evaluation of the parent vessel, iodine and polyenergetic images reconstructed with the Hv56 and Hv72 kernel achieved comparable scores independently of iMAR. For example, polyenergetic reconstructions received a median score of 5 for Hv56 (without iMAR), a median score of 4 for Hv56 with iMAR and a median score of 4 for Hv72. However iMAR-reconstructions of VMI, IOD and PE were not compared separately.

Moderate agreement (α = 0.67) was found between the two readers for qualitative ratings using the 5-point Likert-type scale. The internal consistency of the test, measured by Cronbach’s alpha, was excellent (0.81).

All spectral reconstructions and UHR were affected by IAFD markers in the vicinity of the vessel lumen (IOD-, UHR-, VMI-reconstructions: *p* = 0.005, *p* < 0.001, *p* < 0.001, *p* < 0.001 respectively).

**Fig. 3:**
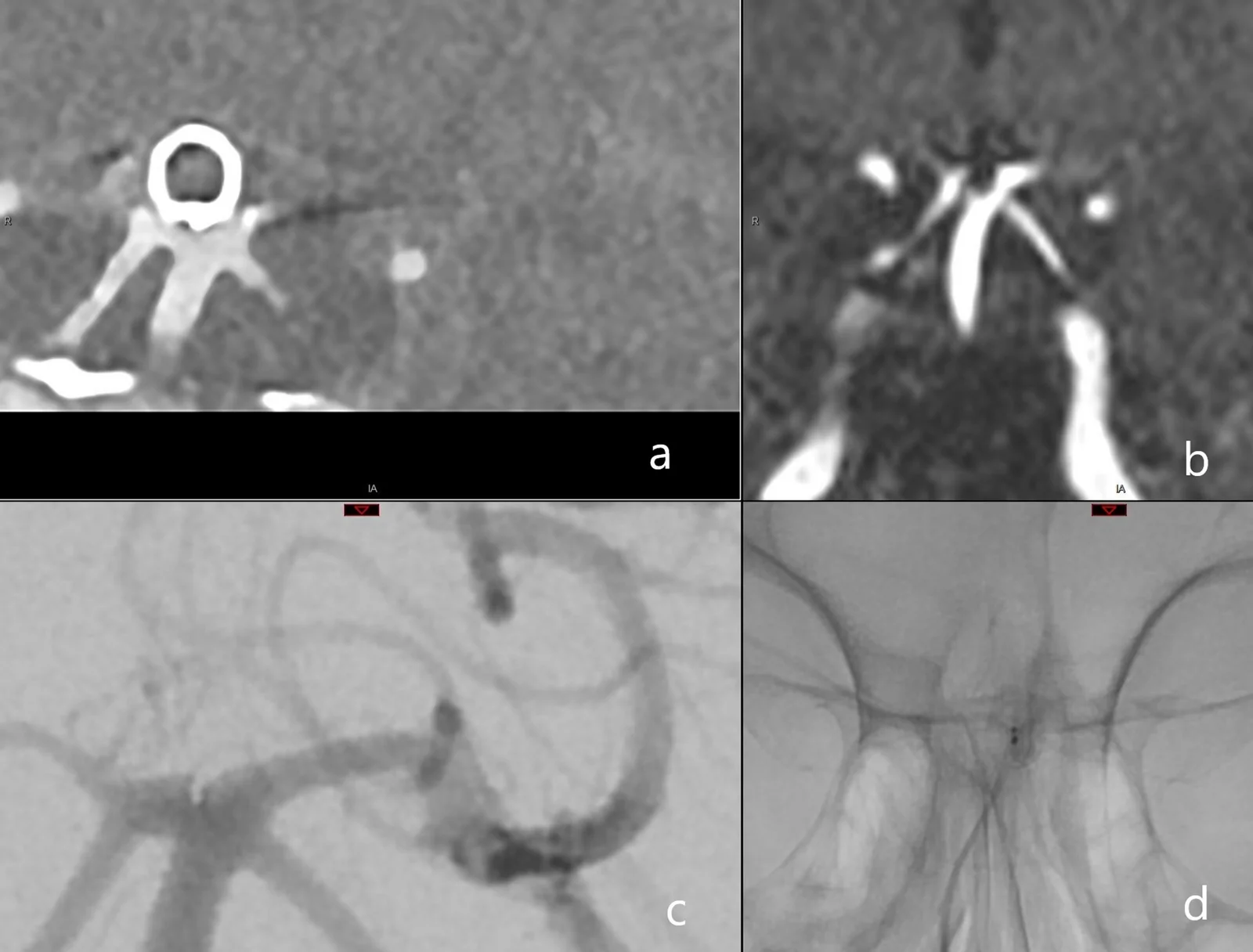
Postinterventional PCD-CTA, TOF-Angiography and DSA of a WEB-device in an aneurysm of the basilar artery. **A** is a coronal VMI reconstruction with Hv 56 at 40keV, **B** is a coronal reformation of a TOF-Angiography, **C** is a p.a. 2D projection of the basilar artery, **D** is a radiography in the same projection. DSA: digital subtraction angiography; Hv: Head vascular kernel, keV: kiloelectron Volt; PCD-CTA: Photon-counting Detector-CT-Angiography; p.a.: posterior-anterior; TOF: Time-of-flight-Angiography; VMI: virtual mono energetic imaging

**Fig. 4:**
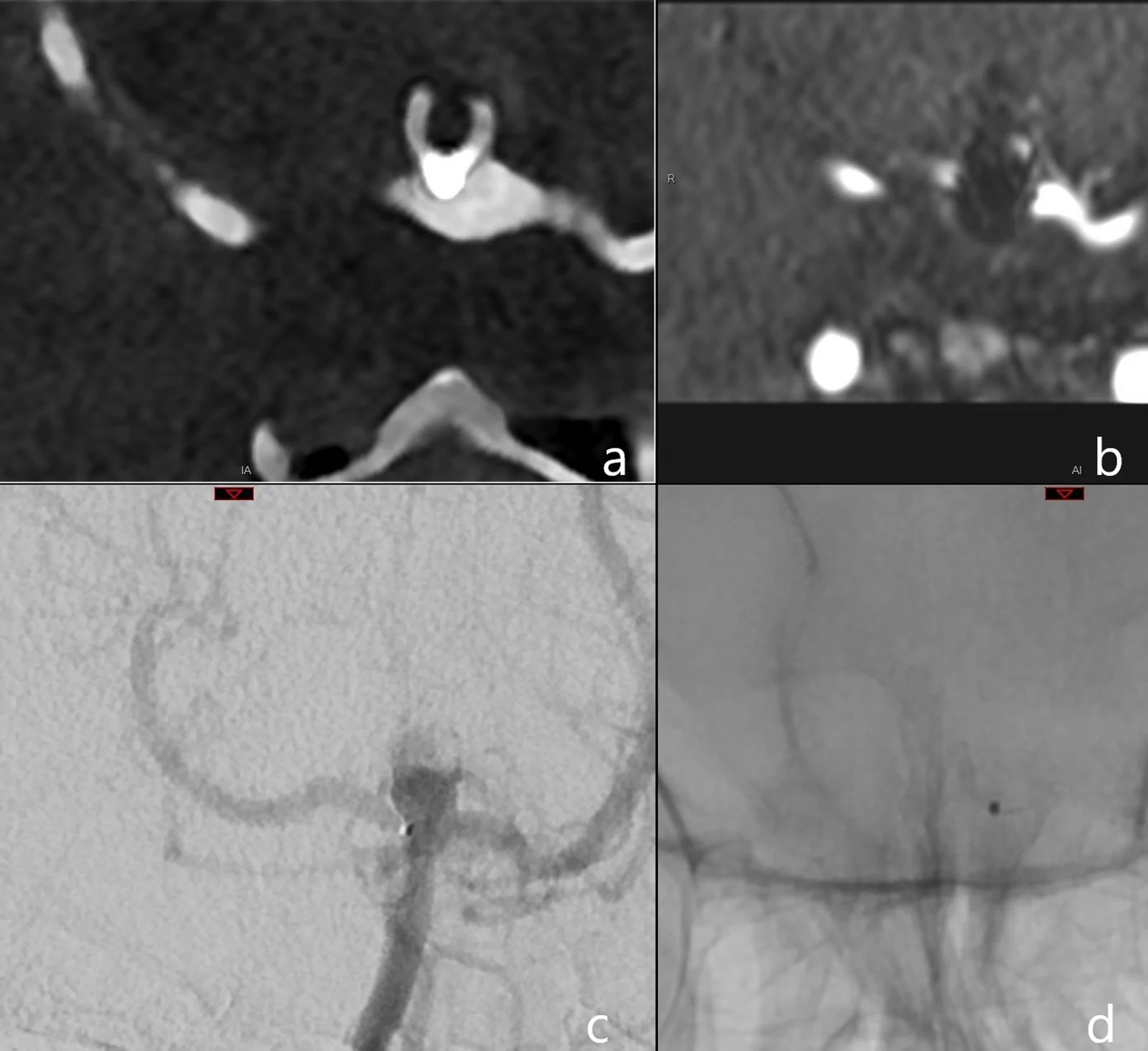
Postinterventional PCD-CTA, TOF-Angiography and DSA of a Contour-device in an aneurysm of the basilar artery. **A** is a coronal VMI reconstruction with Hv 56 at 40 keV, **B** is a coronal reformation of a TOF-Angiography, **C** is a p.a. 2D projection of the basilar artery, D is a radiography in the same projection. DSA: digital subtraction angiography; Hv: Head vascular kernel, keV: kiloelectron Volt; PCD-CTA: Photon-counting Detector-CT-Angiography; p.a.: posterior-anterior; TOF: Time-of-flight-Angiography; VMI: virtual mono energetic imaging

When evaluating the two IAFD separately Contour devices markers produced less artifacts and visualization of the parent vessel only showed minor artifact compared to the WEB-device as visualized in Figs. 3 and 4.

A similar picture to the qualitative assessment also emerged in the quantitative assessment of the best reconstructions for the individual devices. VMI reconstructions with Hv56 and 40 keV showed the highest SNR regardless of the IAFD used.

With a few exceptions [as for example the SNR of the iodine reconstruction using Hv56 had a mean of 7.9 ± 3.2 (mean ± SD) in the Contour device group compared to 4.8 ± 2.1 (mean ± SD) in the WEB-device group (*p* = 0.026)] both the SNR and the CNR showed a similar distribution between the devices.

### Diagnostic performance of PCD-CTA compared with DSA

In patients with implanted IAFD, PCD-CTA provided sufficient image quality to assess aneurysm occlusion and patency of the parent vessel in 9/10 patients.

In one of two patients, who received next to a Contour-device an additional coiling to treat an aneurysm of the left posterior communicating artery, the parent vessel was not identifiable due to artifacts and therefore an aneurysm remnant could not be excluded. A consecutive DSA identified a small aneurysm remnant.

One patient did not receive an additional MRI as PCD-CTA was considered of sufficient quality.

With regard to differences in diagnostic accuracy depending on the IAFD, there were no significant differences between patients with Contour- or WEB-devices when using spectral reconstructions. PCD-CTA provided a high diagnostic accuracy with an AUC of 0.93 (95% CI: 0.77–1).

However, the proximal markers of the Contour device led to well-known susceptibility artifacts in MRI, with consequently limited assessability. TOF-MRA provided a limited diagnostic accuracy with an AUC of 0.67 (95% CI: 0.22–1).

All readers observed a 100% negative predictive value (9/9). Inter observer agreement for diagnostic accuracy was excellent. (κ = 0.86, *p* = 0.004).

## Discussion

This study offers an in-vivo comparison of PCD-CTA with DSA and MRA for evaluating two common intra-aneurysmal flow disruption devices. Reconstructions using smoother kernels and lower keV levels demonstrated superior SNR and CNR outperforming those with harder kernels and higher keV levels.

While current literature points out that neurovascular imaging was enhanced by high spatial resolution, and low keV monoenergetic virtual monoenergetic reconstruction, none addressed specifically intrasaccular devices in-vivo using PCD-CTA [6, 7, 11, 12].

We could reproduce results of a phantom study using flat-panel-CT for evaluating Contour device in a 3D-printed aneurysm model, showing that low-keV reconstructions (40 keV) yielded partial visualization of the aneurysm neck, with persistent artifact issues when using orthopedic metal artifact reduction algorithm [4].

In our study group iMAR algorithm could not enhance the vessel visualization and even altered evaluation of the parent vessel next to the IAFD. Comparable results have been reported with micro-surgical clips [7, 13].

While WEB- and Contour-Device are nitinol-drawn filled tube (DFT) with platinum core wires for improved visibility, they structurally differ not only in the deployed form: Contour has one instead of two markers. Furthermore the Contour marker lies lower in the aneurysm base and closer to the parent vessel [14].

We therefore expected that patients with a WEB device would be more difficult to assess using photon-counting CTA than contour devices, especially in comparison to TOF MRI. However, regardless of the device, PCD-CTA showed better diagnostic performance than TOF MRI. Furthermore, the same reconstructions were rated as best in both subgroups.

### Limitations

Our results lack generalizability concerning the following points:

The patient cohort comprised only a limited number of IAFD overall. Although IAFD markers and subsequent artifacts differed, VMI reconstructions with Hv56 and 40 keV were the best reconstructions regardless of the device, and in all patients with IAFD without coiling and in 1/2 patients with additional coiling, a sufficient assessment of the parent vessel was possible. It remains unclear whether these results can be transferred to other IAFD as the Artisse device. While the best reconstruction parameters were similar in both subgroups, this finding is to be treated with caution due to the limited number of cases.

Even though the true negative value of PCD-CTA is promising, a larger patient cohort is needed to make a more reliable statement regarding diagnostic accuracy in order to be able to dispense with DSA in cases of unremarkable PCD-CTA.

While iMAR altered the parent vessel visualization, this finding is difficult to generalize since our standardized patient positioning with head inclination may certainly consist of a disadvantage for some device locations. Furthermore iMAR-reconstructions of VMI, IOD and PE reconstructions were not compared separately.

Furthermore we did not study systematically the effect of device type and positioning and studied only two kernels and two keV-level, further restricting the generalizability of this finding.

We compared PCD-CTA and DSA only to TOF-MRA. While contrast enhanced MRA proved superior to TOF-MRA in assessing WEB devices, Contour devices are still difficult to evaluate using MRA [2, 4].

Ultra-short echo time MR sequences have been tested in coiled and stent assisted coiled aneurysms. While it seems reasonable to infer they could improve MR based assessment, there is currently no published data available for IAFD [15–17].

## Conclusions

Spectral reconstructions of PCD-CTA with a smoother reconstruction kernel and a low keV level seem to be beneficial to achieve optimal image quality for the diagnostic evaluation of patients with intracranial IAFD.

## Supplementary Information

Below is the link to the electronic supplementary material.

Supplementary Material 1

## Data Availability

No datasets were generated or analysed during the current study.

## Abbreviations

PCD-CTA: Photon-counting detector CT-Angiography
DSA: Digital subtraction angiography
MRA: MR-Angiography
UHR: Ultra-high-resolution acquisition mode
IOD: Iodine reconstruction
VMI: Virtual monoenergetic imaging reconstruction
SNR: Signal-to noise ratio
CNR: Contrast-to noise ratio
keV: Kiloelectron volt
Hv: Head vessel kernel type
SD: Standard deviation
IQR: Interquartile range
