# Supplementary material for: Photon-counting CT-angiography in comparison to digital subtraction angiography and MR-angiography for assessing intra-aneurysmal flow disrupting devices
Source: Acta Neurol Belg. 2026 Jun 30;126(5):1671–9. doi: 10.1007/s13760-026-03112-x (PMC13714051; doi:10.1007/s13760-026-03112-x)
Supplement: Supplementary file 1 [file 13760_2026_3112_MOESM1_ESM.docx]

**Scanning protocol**

Patients underwent a CTA centered on the implanted device acquired on a clinical first-generation PCD-CT scanner (NAEOTOM Alpha, Siemens Healthineers, Erlangen, Germany) operated in ultra-high-resolution (UHR) mode, resulting in a reconstructed slice thickness of 0.2 mm and slice increment of 0.1mm. Spectral reconstructions were reconstructed in 0.4 mm slice thickness and 0.2 mm slice increment. The reconstructed matrix size was 1024 x 1024, and the field of view was adjusted for each patient to optimally image the intraaneurysmal flow disrupting device.

The following acquisition parameters were used: tube voltage 140 or 120kvp, pitch 0.65, rotation time 0.5 s. CTA was performed after the administration of 80 ml iodinated contrast material (Ultravist-370 (generic name, iopromide; Bayer Healthcare, Berlin, Germany), injected through a 20-gauge intravenous antecubital vein catheter using a power injector. The flow rate 4ml/s. Opacification of the common carotid artery was monitored using a bolus tracking technique. The start time of data acquisition was determined with a fixed delay of five seconds after the attenuation threshold was reached. Iterative reconstruction (denoted “QIR” by the manufacturer) level 3 was used for all PCD-CTA images. Polyenergetic reconstructions in UHR-mode as well spectral reconstructions were performed for Iodine, Virtual monoenergetic images (VMI). Two image sets were reconstructed using the following vascular kernels: Hv56, Hv72. For VMI-Reconstructions two keV levels (40, 80) were reconstructed for each kernel. The CT images with the Hv56 kernel were reconstructed using iterative Metal Artifact Reduction (iMAR) for each energy level (40 and 80 keV).
